# A Polyendocrine Puzzle: Unravelling Schmidt Syndrome (Autoimmune Polyendocrine Syndrome Type II) Presenting in Crisis

**DOI:** 10.7759/cureus.94800

**Published:** 2025-10-17

**Authors:** Shadman Sakib Rahman, Nusrat A Chowdhury, Nandakumar Poonthottam, Sumaiya Kamal, Mohammad Arif Sarwar

**Affiliations:** 1 Internal Medicine, Medway NHS Foundation Trust, Kent, GBR; 2 Acute Medicine, Medway Maritime Hospital, Gillingham, GBR; 3 Acute Medicine, Medway NHS Foundation Trust, Kent, GBR

**Keywords:** addisonian crisis, addison’s disease, autoimmune disorder, autoimmune polyendocrine syndrome type ii, autosomal dominant inheritance, graves’ disease, hydrocortisone, primary adrenal insufficiency, short synacthen test, steroid

## Abstract

Schmidt syndrome is a rare autoimmune disorder with polygenic inheritance that results in damage to specific organs due to lymphocytic infiltration. It is characterised by the coexistence of at least two of the following: Addison’s disease, autoimmune thyroid disease (Graves’ disease or hypothyroidism) and type 1 diabetes mellitus (T1DM). We report such a rare case, in which a 34-year-old woman presented with symptoms of adrenal insufficiency and was diagnosed with Schmidt syndrome. Prompt recognition, patient education and long-term multidisciplinary follow-up are essential for optimal management of such conditions. Empirical glucocorticoid therapy before a definitive diagnosis was crucial in the management of such a crisis. This case highlights the rarity and diagnostic challenges of this syndrome, in which nonspecific symptoms, along with overlapping autoimmune conditions, may delay diagnosis and subsequent management.

## Introduction

Schmidt syndrome (autoimmune polyendocrine syndrome (APS) type II) is a rare autoimmune disorder that can be described as a single patient presenting with more than one endocrine disease [1]. At least two of the three specific disorders (Addison’s disease, autoimmune thyroid disease (Graves’ disease or hypothyroidism) or type 1 diabetes mellitus (T1DM)) must be present to meet the diagnostic criteria [1,2]. The estimated prevalence stands at only 1.4-4.5 per 100,000 population, being more common in middle-aged women and rare in children [3,4]. The aetiology primarily involves lymphocytic infiltration leading to organ damage [1]. Compared to patients with Addison’s disease, there is a 2.5-fold higher risk of crisis in those with APS type II [1].Therefore, treating physicians should involve a multidisciplinary team led by an endocrinologist with routine follow-up to ensure early diagnosis, provide patient education, prevent complications and achieve optimal patient care.

## Case presentation

**Figure 1:**
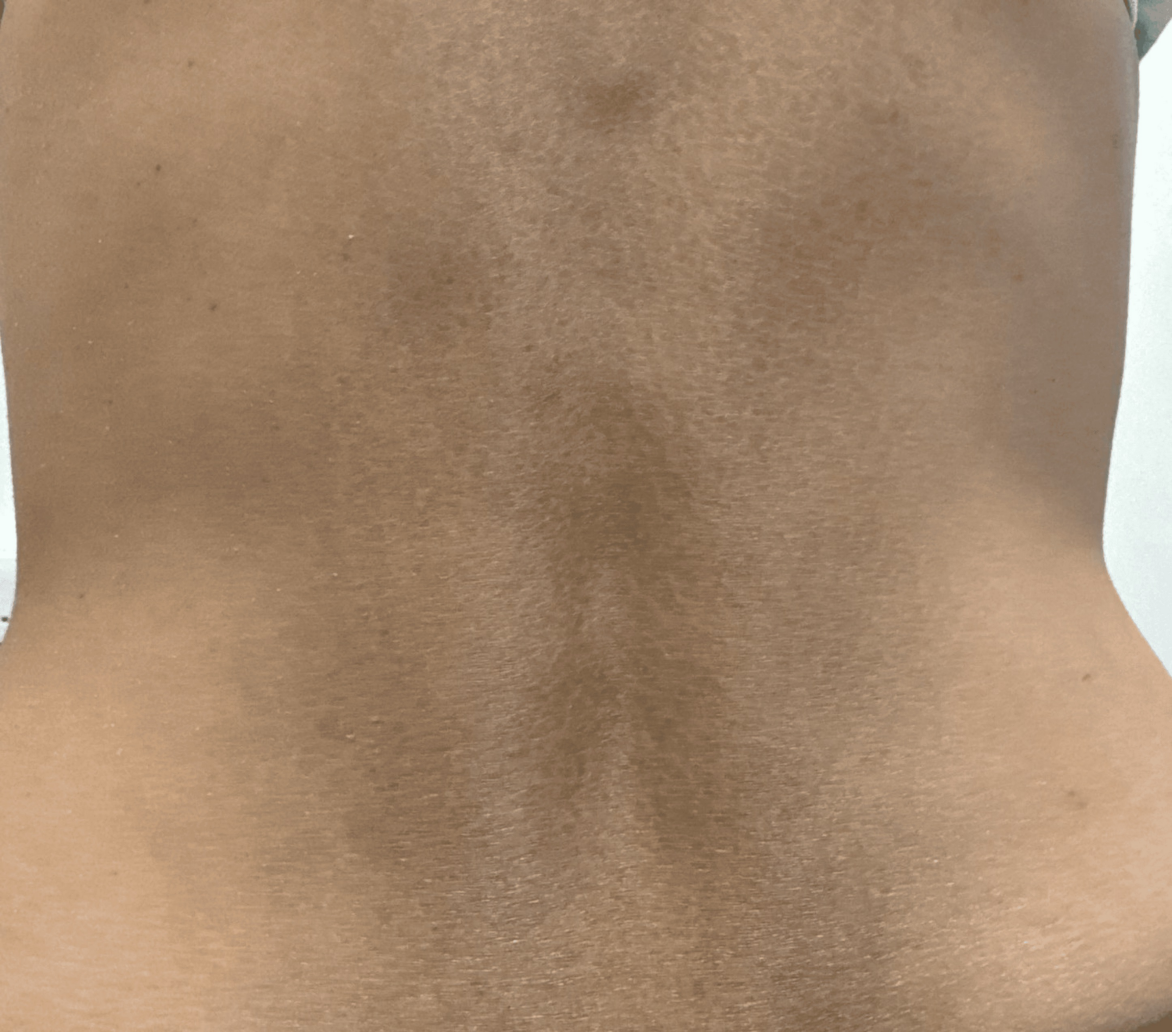
Hyperpigmentation of the lower back

We report the case of a 34-year-old White British lean and thin woman who presented to the hospital after referral from her general practitioner (GP) with very low sodium levels (Na: 122 mmol/L), along with mildly raised potassium (K: 5.3 mmol/L). On questioning, she mentioned a six-month history of fatigue, salt cravings and shortness of breath and noticed increasing pigmentation of the skin on her back (Figure 1). She has had a medical background of only Graves’ disease for 10 years, but became hypothyroid following radioiodine therapy, and she is now on levothyroxine replacement therapy. She mentioned that her father also had Addison’s disease. On examination, hyperpigmentation was noted over the dorsum of her hands (knuckles) and across her lower back (Figure 1). She was hypotensive with a blood pressure of 85/60 mmHg. A provisional diagnosis of adrenal insufficiency was considered, and routine blood tests (Table 1) were requested along with diagnostic investigations (Table 2) on admission.

**Table 1:** Relevant routine blood investigations on admission TSH: thyroid-stimulating hormone, CRP: C-reactive protein, INR: international normalised ratio, eGFR: estimated glomerular filtration rate, BNP: brain natriuretic peptide

Parameter	Value	Normal range
Sodium (mmol/L)	122	133-146
Potassium (mmol/L)	5.3	3.5-5.3
TSH (mIU/L)	4.3	0.3-4.8
Vitamin D (nmol/L)	36	>50
Amylase (IU/L)	169	28-100
Plasma BNP (pg/mL)	108	0-99
CRP (mg/L)	0.3	0-5
Haemoglobin (g/L)	136	120-150
INR	1	0.8-1.2
eGFR (mL/min)	75	>90
Creatinine (umol/L)	77	45-84
Urea (mmol/L)	7.7	2.5-7.8

**Table 2:** Diagnostic investigations ACTH: adrenocorticotropic hormone, IGF-1: insulin-like growth factor 1, FSH: follicle-stimulating hormone, LH: luteinising hormone

Parameter	Value	Normal range
HbA1C (mmol/mol)	34	20-41
9 am cortisol (nmol/L)	146	185-624
ACTH (ng/L)	>2,000	7.2-63.3
Plasma renin (mU/L)	593.9	3.4-56
Aldosterone (pmol/L)	<50	100-450 (supine)
Short Synacthen test	Positive	
Adrenal cortex antibodies	Positive	
30-minute cortisol (nmol/L)	142	>450
60-minute cortisol (nmol/L)	127	>450
Serum IGF-1 (nmol/L)	14.4	9.0-33.8
FSH (IU/L)	5.8	2.5-10.2
LH (IU/L)	4.1	1.9-12.5
Prolactin levels (mIU/L)	215	70-566
Coeliac screen	Negative	

Management 

While awaiting diagnostic investigations, treatment was initiated empirically for suspected adrenal insufficiency with intravenous (IV) hydrocortisone, proton pump inhibitors (PPI) and bone protection. She received 500 mL of normal saline (0.9% NaCl) over two hours initially, followed by 1,000 mL every eight hours for 24 hours to improve her blood pressure and hydration status. After she became clinically stable with IV steroids and fluids, she was discharged on oral hydrocortisone tablets (20, 10 and 10 mg) and fludrocortisone 100 mcg tablets, along with an ambulatory clinic follow-up for pending bloods to be reviewed and discussed with the endocrine specialist.

Ongoing review in ambulatory care of her blood results (low cortisol, low aldosterone and raised adrenocorticotropic hormone (ACTH)) prompted a short Synacthen test, which was positive (Table 2). She also had positive adrenal cortex antibodies, and other autoimmune screens, including a coeliac screen, were negative. Based on these laboratory findings, a diagnosis of Addison’s disease was made. This clinical condition, along with her previous medical background of Graves’ disease, led to a diagnosis of APS type 2 (Schmidt syndrome).

The patient was educated about the condition and provided with safety netting advice. An emergency intramuscular (IM) kit was provided, and the sick day rule (doubling the steroid dose) was explained to the patient and her partner. A steroid bracelet and card were given.

**Figure 2:**
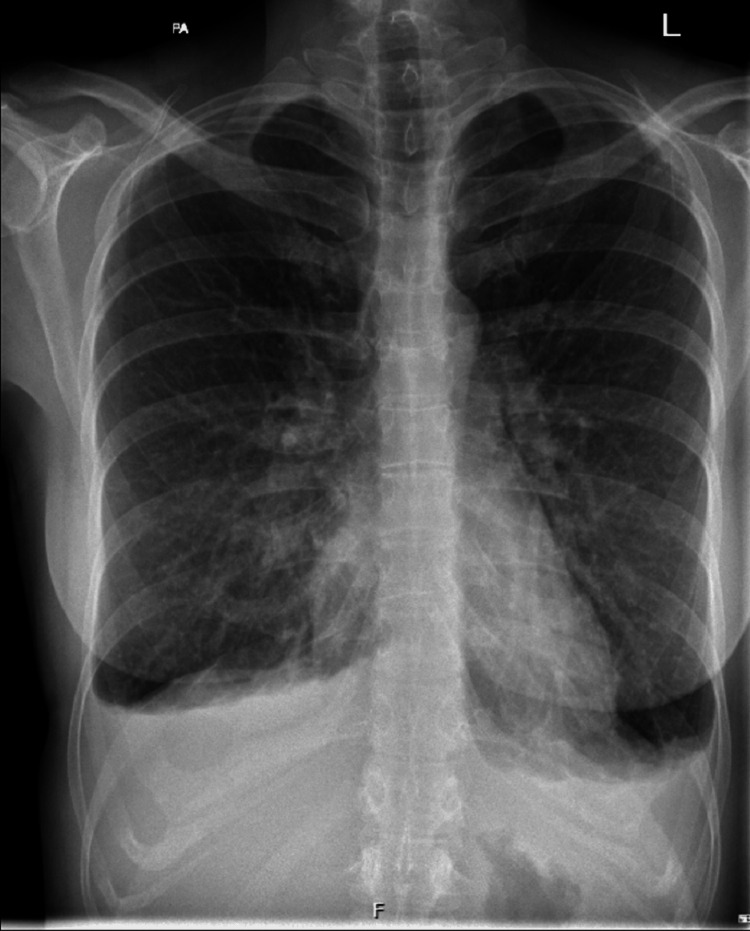
Chest X-ray showing bilateral pleural effusion

**Figure 3:**
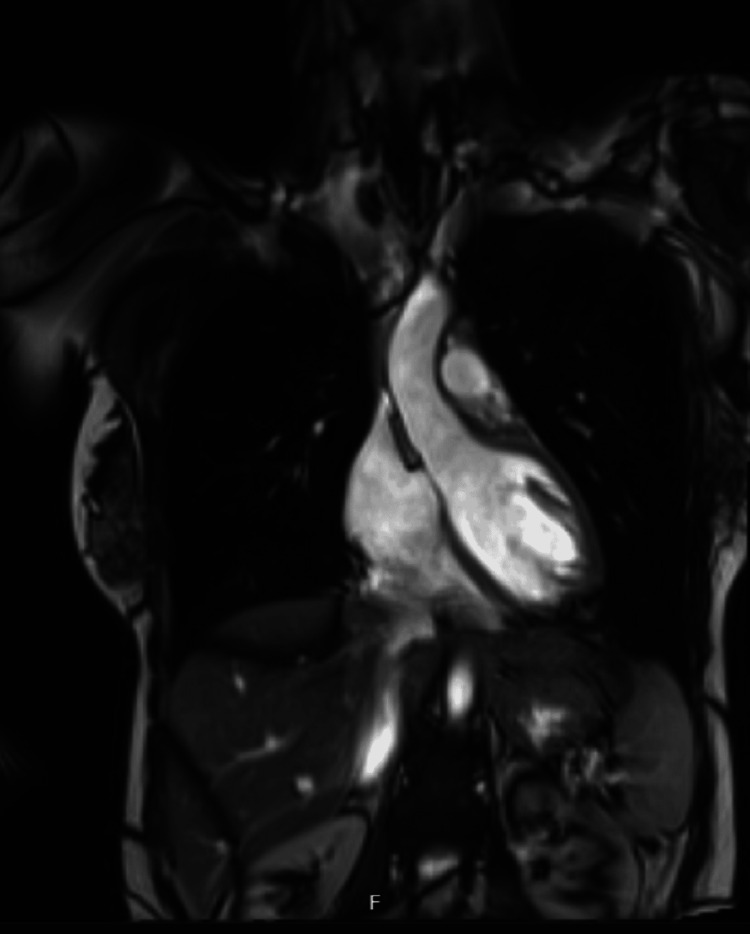
Cardiac MRI (coronal section): normal LVOT MRI: magnetic resonance imaging, LVOT: left ventricular outflow tract

She was routinely reviewed in ambulatory care to optimise her steroid medications and monitor her renal function when, on one occasion, she developed signs and symptoms of fluid overload. On examination, there were bilateral crackles on auscultation of her chest and pitting oedema of the legs. Following these findings, a chest X-ray was performed, which showed bilateral pleural effusion (Figure 2). Brain natriuretic peptide (BNP) was also measured and found to be raised. Fludrocortisone was suspended due to concerns about fluid overload. An echocardiogram was requested, which did not have any significant findings, and the left ventricular ejection fraction was more than 55%. Her symptoms gradually improved following suspension of fludrocortisone. She was subsequently followed up in the Cardiology Hot Clinic, and the team requested a cardiac magnetic resonance imaging (MRI) as surveillance for cardiomyopathy or structural heart disease. In the meantime, her hydrocortisone dose was tapered to 10, 5 and 5 mg, and a lower dose of fludrocortisone 50 mcg was restarted. The cardiac MRI did not show any significant findings (Figure 3).

**Figure 4:**
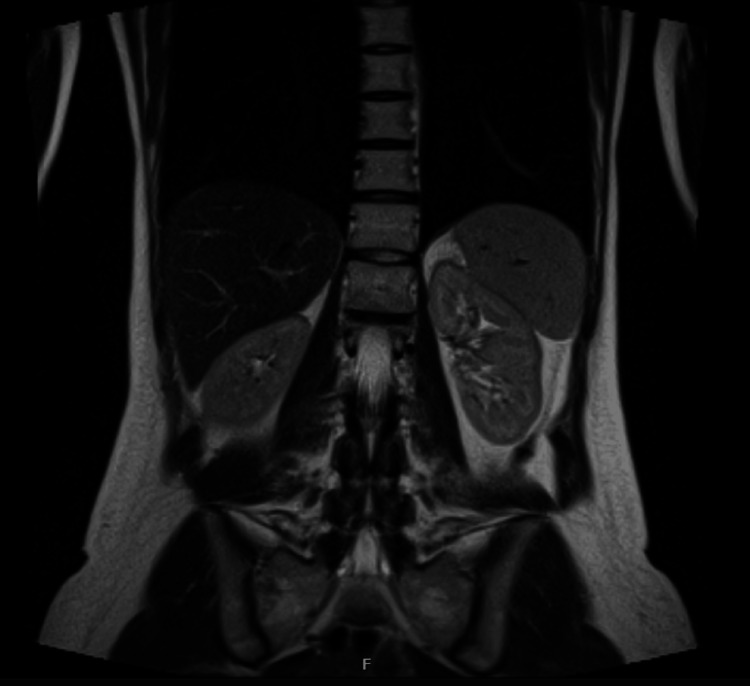
MRI of the bilateral adrenal gland (coronal section): no adrenal pathology MRI: magnetic resonance imaging

She is currently under the care of the endocrine team and is reviewed regularly in their clinic. She had an MRI of her adrenal glands to assess for any adrenal pathology. The MRI revealed no significant abnormalities (Figure 4).

Follow-up

She is now well aware of her diagnosis and knows how to respond if she develops any symptoms of crisis. She will have routine endocrine clinic appointments for review of her blood tests and is now maintained on stable doses of hydrocortisone and fludrocortisone with no further deterioration of her symptoms. She feels well supported by her medical team and is compliant with her medications.

## Discussion

This condition poses significant diagnostic challenges due to the nonspecificity of the clinical features [2]. Given the risk of acute deterioration following crisis due to various precipitants, prompt and correct diagnosis followed by suitable hormone replacement therapy is often critical to patient survival [2]. A holistic approach to management is also essential to avoid over- or under-treatment [1].

Primary adrenal insufficiency is due to an autoimmune process that destroys the adrenal cortex. When both cell-mediated and antibody-driven immune responses target the adrenal cortex, it is often associated with autoimmune destruction of other endocrine glands as well. This is called polyglandular autoimmune (PGA) syndrome [4]. There are three main types of PGA: PGA I, PGA II and PGA III. PGA I comprises mucocutaneous candidiasis, hypoparathyroidism and adrenal insufficiency. PGA II or Schmidt syndrome involves at least two of the following three: adrenal insufficiency, autoimmune thyroid disease and type 1 diabetes. PGA III comprises only thyroid autoimmunity and type 1 diabetes [5].

Schmidt syndrome is a polygenic disorder inherited in an autosomal dominant pattern with incomplete penetrance and is linked to the following genes: *HLA-DR3*, *HLA-DR4*, *CTLA-4*, *PTPN22* and *CD25-IL-2* [5-7].At present, tuberculosis causes only about 7%-20% of Addison’s cases, whereas autoimmune conditions account for the majority, around 70%-90%. The remaining cases are caused by other infectious diseases, metastatic cancer, lymphoma and adrenal haemorrhage or infarction [4]. In developed countries, autoimmune adrenalitis causes about 75%-80% of Addison’s disease cases, while tuberculosis accounts for 7%-20% and remains the leading cause in developing nations [8].In Schmidt syndrome, autoimmune thyroid disease occurs in about 70%-75% of cases, type 1 diabetes mellitus in 40%-60% and Addison’s disease in roughly 40%-50% [9].

Morbidity and mortality in Addison’s disease can be greatly reduced by early diagnosis. However, the disease can be insidious, and the symptoms can be nonspecific, which often complicates and delays the diagnosis [10-12]. The diagnosis of Schmidt syndrome requires a lifelong commitment from the patient and requires regular medication, with appropriate dose adjustments during periods of stress or illness to prevent complications [13]. We also emphasise that regular long-term follow-up of these patients is essential [14,15].

## Conclusions

Initiating treatment early, rather than waiting for a formal diagnosis, played a crucial role in ensuring safe medical practice, as there was a risk of acute deterioration, given that the patient was already on thyroxine replacement, which could precipitate an adrenal crisis. Therefore, when both cortisol and thyroid levels are low, glucocorticoids must be given and wait for 24-48 hours or stabilising the patient before safely initiating thyroxine treatment. Another challenge was managing the transient mineralocorticoid-driven fluid overload symptoms that developed in the patient. Requesting all appropriate investigations is also vital to establishing the diagnosis and screening for other autoimmune conditions. Given the rarity of the condition, most patients would present with limited knowledge about the condition and should be made aware that it is a lifelong condition requiring routine monitoring, so that they can effectively incorporate its management into their lifestyle.
